# Patient-pharmacist communication during a post-discharge pharmacist home visit

**DOI:** 10.1007/s11096-018-0639-3

**Published:** 2018-05-02

**Authors:** Hendrik T. Ensing, Marcia Vervloet, Ad A. van Dooren, Marcel L. Bouvy, Ellen S. Koster

**Affiliations:** 1 Research Group Process Innovations in Pharmaceutical Care, Utrecht University of Applied Sciences, Utrecht, The Netherlands; 2 Department of Pharmacoepidemiology and Clinical Pharmacology, Utrecht Institute for Pharmaceutical Sciences (UIPS), Utrecht University, Utrecht, The Netherlands; 3 Zorggroep Almere, Outpatient Pharmacy “de Brug 24/7”, Hospitaalweg 1, 1315RA Almere, The Netherlands; 4 NIVEL, Netherlands Institute for Health Services Research, Utrecht, The Netherlands

**Keywords:** Community pharmacist, Continuity of care, Home visits, Hospital discharge, Patient-provider communication, Seamless care, The Netherlands, Transitions of care

## Abstract

*Background* With the shifting role of community pharmacists towards patient education and counselling, they are well-positioned to conduct a post-discharge home visit which could prevent or solve drug-related problems. Gaining insight into the communication during these home visits could be valuable for optimizing and consequently improving patient safety at readmission to primary care. *Objective* To assess patient-pharmacist communication during a post-discharge home visit. *Setting* The homes of patients recently discharged from a single general hospital in the Netherlands. *Methods* Pharmacists used a semi-structured protocol to guide the consultations and audiorecorded them. Sixty audio-recordings were included for a qualitative analysis in this study with the help of NVivo version 11 software. *Main outcome measure* (1) Initiator and topics under discussion. (2) Frequency of discussion of topics as per coded in themes and subthemes. *Results* Issues regarding the administration and use of medication, e.g. regimen and actual drug-taking issues, knowledge gaps regarding their medication and patients’ health were discussed most frequently, followed by medication logistics and medication effectiveness. Patients’ beliefs about their medication and adherence were less frequently discussed. The pharmacist initiated the majority of these topics. Additional non-protocolled topics were scarce and consisted mainly of patient-initiated dissatisfaction regarding the community pharmacy or health insurers. *Conclusion* Community pharmacists most frequently initiated practical issues, but explored patients’ medication beliefs less adequately. Discussing these beliefs might be easier by increasing patient engagement in the consultation and providing training programs for pharmacists.

## Impacts on practice

- A home visit protocol enables pharmacists to address known major challenges during the transition from hospital to primary care
- Addressing patient’s dissatisfaction about health care is important as it facilitates patient participation during consultation and acceptance of pharmacists’ advices
- Pharmacists should discuss patients’ medication beliefs and adherence issues more frequently, which might be facilitated by additional pharmacist training and increasing patient engagement

## Introduction

The community pharmacist’s role is shifting from traditional medication dispensing to patient education and counselling [1]. Patient transition from hospital back to their home provides pharmacists with the opportunity to effectuate this role, as this transition is associated with an increased risk of drug-related problems (DRPs). Inadequate patient counselling during the transition is a contributing factor [2, 3]. Pharmacists are well positioned to facilitate the discharge process by performing medication reconciliation, identifying patients with poor health literacy or non-adherence, and providing tailored discharge counselling [4]. However, to establish continuity of care most efficiently and provide adequate patient support, discharge procedures should be complemented with adequate post-discharge follow-up [5]. Introducing a post-discharge community pharmacist home visit can secure continuity of care but is not usual care at the moment in the Netherlands.

Community pharmacists must adapt their communication to address the wide variety of patients’ drug-related problems during these home visits and achieve patient-centred communication. Patient-centred communication is associated with increased patients’ satisfaction, better recall of information and improved health outcomes and requires active participation of both the pharmacist and the patient [6–8]. Patients should be encouraged to express their needs and concerns regarding their medication, which pharmacists should address to support patients in making informed decisions [9]. Little is known about the topics discussed during a post-discharge home visit and most studies investigating patient-pharmacist communication focused primarily on one-way pharmacist information provision, e.g. the extent to which pharmacists counsel patients, and their communication style, e.g. tone of voice [10, 11]. Gaining insight in the communication during these home visits could be valuable for optimizing these visits; and consequently to improve patient safety at readmission to primary care.

## Aim of the study

To assess patient-pharmacist communication during a post-discharge home visit by exploring the discussed topics as well as who—the patient or the pharmacist—initiated a specific topic.

## Ethics approval

Ethical approval was obtained from the ethics committee of the Radboud University Medical Centre Nijmegen. Local approval was obtained from the scientific committee of Zorggroep Almere (ZGA, Care Group Almere) and Flevoziekenhuis Almere. Patients gave written informed consent at inclusion and oral consent for audio recording of the consultation at the start of the home visit. All data files were coded by using unique personal identification numbers and personal details were removed from the transcripts.

## Method

### Study setting

A qualitative observational study was conducted with audio-recordings from community pharmacist home visits from the Home-based Community pharmacist-led Medication management (HomeCoMe) program that were performed between November 2013 and December 2014 [12]. The in-hospital outpatient pharmacy acted as a discharge coordinator and cooperated closely with all community pharmacists. It verified patients’ administrative information, reiterated important study information, notified the community pharmacists of a pending discharge and transferred all medication-related information to them.

The HomeCoMe program consisted of in-hospital pharmacy interventions and its main component: a post-discharge home visit by the patient’s own community pharmacist [12]. Pharmacists used a semi-structured protocol to address patients’ questions and reinforce medication-related hospital discharge information. Furthermore, pharmacists aimed to identify and solve pending and emerging post-discharge drug-related problems (DRPs) during the home visits by (1) performing post-discharge medication reconciliation, (2) assessing patients’ medication knowledge, (3) identifying adherence barriers and (4) determining patients’ concerns [12]. Deploying home visits instead of a telephone follow-up is possibly more beneficial due to the personal touch of face-to-face encounters [13]. Patients might feel more comfortable at home and are therefore more likely to share their experiences and concerns about their medication and be more receptive to pharmacist’s counselling. Furthermore, a home visit may elicit all relevant DRPs since all medication is available at home enabling the assessment of specific risk factors, such as inappropriate medication storage conditions [14].

### Study population

Patients were eligible if they were discharged from a single general hospital (neurology and pulmonology wards) to their own home, aged 18 years or over, used at least three or more prescription drugs for chronic use at discharge, had been hospitalized for at least 48 h and picked up their medication in one of the participating pharmacies.

### Pharmacist home visit protocol

The community pharmacists contacted the patients as soon as possible post-discharge and aimed to visit them within 7 days. A semi-structured protocol to guide pharmacists during the home visits was used (Table 1). Efforts were made to develop a protocol tailored to the individual patient by: (1) assessing patients’ perceptions on their use of medication in general and specifically for medication started during hospitalization and (2) incorporating open-ended example questions, e.g. for initiating and finalizing the home visit. These efforts aimed to help pharmacists to focus on problems relevant to the patient [10]. All participating community pharmacists previously attended accredited courses on performing medication reviews, including patient interviews. To ensure generalizability, all pharmacists received an additional one-day training course on how to perform the home visit and how to tailor their communication to the needs of the individual patient. Besides plenary instructions, the pharmacist practiced with the home visit protocol with the help of paper patients and role-playing.

**Table 1:** Main topics to be addressed during the post-discharge home visits

Protocol part	Aim
Introduction	To list the topics that the patient wants to discuss, set the patient at ease and clarify the aim of the home visit
Clinical issues	To obtain an overall impression of patient’s health. This part contains a checklist of possible (drug-related) health issues and example questions to address these issues
Beliefs about medication	To clarify patient’s beliefs and concerns about medication, their attitude towards taking medication, the (lack of) effect of their medication, experienced side effects and intentional adherence barriers
Practical issues	To clarify patient’s practical issues with their medication, e.g. difficulties adhering to their daily regimen, with the packaging, with the actual drug taking or unintentional adherence barriers such as forgetfulness or lack of stock
Patient’s knowledge	To identify patient’s knowledge gaps concerning their medication, e.g. reason for prescribing, medication regimen, duration of use and administration of medication
Spare medication	To identify and collect possible spare medication
Conclusion	To conclude the home visit by ensuring the patient has discussed all his topics, summarize and solve identified (drug-related) problems and provide patient with information on the follow-up

### Data collection

In total, 152 patients received a post-discharge home visit, which was audio-recorded by the community pharmacists. Incomplete recordings or recordings with very poor sound quality were excluded. This resulted in 122 recordings (78.9%) eligible for inclusion of which a random sample of 60 recordings was selected for this study. No new subthemes were identified after 30 recordings, therefore this most likely ensured data saturation. At least one recording from 23 of the 26 participating community pharmacists was included. The recordings from the other three pharmacists were incomplete. To complete data selection a pragmatic approach was used to obtain a selection of recordings that were equally distributed on pharmacists’ gender, patients’ gender and the presence or absence of an informal caretaker during the home visit.

### Data coding and analysis

Two research-assistants transcribed all 60 recordings verbatim to ensure consistency. All transcripts were imported into NVivo version 11 software to facilitate analysis.

All transcripts were coded and reviewed by a researcher (HE) and a research assistant (LV). Discrepancies were resolved through discussion and, if necessary, a third researcher (MV) was consulted to reach consensus. A thematic content analysis was used to examine main themes [15]. First, the three overarching themes based on the HomeCoMe protocol were identified: (1) ‘Medication”, (2) “Clinical” and (3) “Other” (Table 2). Next, all subthemes were coded inductively. After coding of the first five transcripts these subthemes were redefined and merged where possible into a preliminary codebook. Previously coded transcripts were re-coded to match any changes in theme definitions during this coding procedure. During coding of the remaining transcripts a process of reading and re-reading, with attention to the identification of new subthemes, eventually resulted in the final code book with well-defined codes and descriptions (Table 2). Additionally, the initiator of each subtheme was coded (pharmacist or patient) as well as an illustrative quote.

**Table 2:** Condensed codebook displaying themes, subthemes and examples

Theme	Subtheme (example)
Medication	Medication information (e.g. indication, side effects, mechanism of action)
	Medication effectiveness (e.g. perceived effect of medication)
	Non-prescription medication (e.g. over-the-counter medication, vitamins)
	Beliefs about medication (e.g. needs, concerns, usefulness of medication,)
	Medication logistics (e.g. repeat prescription issues, stock issues)
	Medication adherence (e.g. practical or perceptual adherence barriers)
	Administration and use (e.g. actual drug-taking, medication regimen, multi-dose dispensing system)
Clinical	Patients’ general health (e.g. existing health issues, worsened symptoms)
	Hospital admission (e.g. reason for hospitalization, length of stay)
Other	All themes unrelated to the HomeCoMe protocol (e.g. satisfaction with health care providers, personal information)

All data was descriptively analysed by identifying major themes, based on frequency of being mentioned, and the initiator of those themes.

## Results

### General characteristics

The 23 pharmacists had a mean of 17.7 ± 8.3 years of working experience in the community pharmacy and performed a mean of 6.5 ± 5.6 home visits. The 60 audio-recordings lasted 28.4 ± 11.4 min on average.

The mean age of the patients was 65.3 ± 13.5 years and 51.7% were females. A partner or informal carer was present during 20 home visits (33.3%).

### Patient: pharmacist communication

**Fig. 1:**
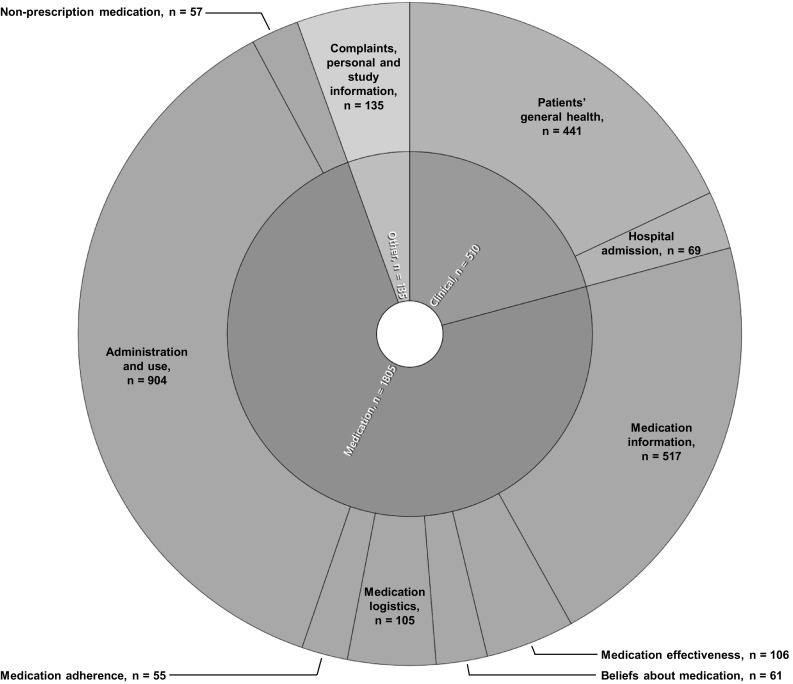
Distribution of themes (inner circle) and subthemes (outer circle). In total, 2450 text fragments were coded

In total 2450 text fragments were coded (Fig. 1). Approximately three-quarters of the topics discussed during the home visits can be classified within the theme “Medication”, followed by “Clinical” topics. Only a few additional topics were classified within the “Other” theme (Fig. 1). The five major subthemes, ranked by frequency, and accompanying illustrative quotes are described in more detail below, as well as less-discussed subthemes and topics in the “Other” theme.

#### Administration and use

Administration and use was the largest subtheme (Fig. 1). The majority of topics within this subtheme concerned patients’ medication regimens which pharmacists initiated more often than patients. Pharmacists identified possible knowledge gaps and reinforced the information concerning patients’ discharge medication regimens, explored and advised on possible regimen improvements, clarified the duration of use for temporary medication (e.g. pain medication started at discharge) or determined patients’ daily regimens for medication with an alternating dose schedule (e.g. insulin).

*Pharm5: “We’ll discuss the medication that is discontinued during hospitalization in just a moment.” Pat10: “Yeah, there are a lot of them!” Pharm5: “That’s right, let’s discuss them one by one.”*

*Pharm19: “All right, let’s see, do you have any questions regarding the use of your medication? Pat54: “Well, I’m familiar with most of them, but I have some questions about those two new inhalers.”*

Patients initiated topics in this subtheme to clarify uncertainties concerning their regimen (e.g. questions about medication changes) and to ask for advice.

*Pat20: Yes, it’s very convenient that you’re here. I was discharged from the hospital last Wednesday and there are two medicines I had before which I did not receive at my discharge. Should I still take them?”*

Potential drug-taking issues were explored more often by pharmacists than patients. Pharmacists gathered information for instance on any discomfort with taking the medication, and consequently evaluated the relevance and provided advice or support.

*Pharm18: “Could you show me how you use your spacer?” Pat50: “Yes, I have got this blue one. It should not whistle, as that indicates that I am inhaling too fast.”*

If patients initiated drug-taking issues, they shared their experiences, or asked for advice to solve drug-taking issues.

*Pat15: “You’re supposed to dissolve these [amoxicillin] in water, but well, I’ve skipped that sometimes. I did it whenever I could though.” Pharm6: “That’s ok, you can also take them without previously dissolving them.”*

Other less frequently discussed topics concerned packaging (e.g. opening blisters), multi-dose dispensing systems or receiving support (e.g. from partner) in taking their medication.

#### Medication information

The majority of topics within the “Medication information” subtheme concerned the indications for use. Pharmacists explored this topic more often than patients and informed patients about the reason for prescribing specific medication.

*Pharm21: “Can you tell me why you have to take these [diclofenac]?” Pat57: “Sure, I have to take those three times a day. It’s an anti*-*inflammatory drug and a painkiller as well.”*

If patients initiated this topic they indicated to be unaware of the reason for prescribing, mainly in cases of using multiple medications.

*Pat32: “Is that the one to reduce my cholesterol levels?” Pharm12: “No, these prevent your blood from clotting.”*

Topics related to side effects were initiated more often by pharmacists than patients as well. Pharmacists verified patients’ knowledge on medication side effects, checked if patients experienced a side effect, acknowledged the existence of a side effect or reassured the patient.

*Pharm23: “You’re using a fairly high dosage of bisacodyl, do you experience any side effects like stomach ache or nausea? Pat59: “No, not at all.”*

Patients initiated this topic to share information about experienced side effects.

*Pat5: “If I use them, I continuously have to go to the toilet, I really hate that!” That’s why I skipped a dosage today. Thursday I have to take another one and it all will start again.” Pharm2: Okay and did you experience any adverse effects from skipping that dosage, for instance shortness of breath or fluid retention in your legs? Pat5: “No not at all, but my specialist warned me that I should really take them.”*

Other topics within this subtheme were discussed less frequently and concerned the mechanism of action of the medication and any precautions (e.g. driving precautions).

#### Patients’ general health

Pharmacists initiated topics concerning patients’ general health more often than patients. They queried patients using a trigger list on possible existing health issues including follow-up (e.g. laboratory tests or GP-visits), inquired for worsened or improved symptoms post-discharge or provided life-style advice (e.g. smoking cessation or exercise).

*Pharm1: “You were admitted for meningitis, how are you doing right now?” Pat2: “Reasonably.” Pharm1:”You’re not left with any lingering symptoms?” Pat2: “Well yes, I experience some rigorous shaking, especially during physical exercise.”*

If patients took initiative, they shared information on experiencing a specific health issue.

*Pat33: “Well, to be honest, the tumour affects my breathing. I experience shortness of breath, but luckily I’m not in pain.”*

#### Medication logistics

Patients participated more actively within this subtheme, however pharmacists initiated topics on medication logistics still more often than patients. Pharmacists verified patients’ medication stock, elucidated and advised on storage conditions and on obtaining repeat prescriptions and collected discontinued or expired medication.

*Pharm8: “Does it sometimes happen that you don’t have enough medication left?” Pat22: “No, not at all! My wife and I pay really good attention to having an adequate stock at home.”*

Patients initiated these topics mainly to gather information or to share their supply inconveniences.

*Pat24: “Where and how do I get my prescription for those pills? Should I contact the specialist or the GP?”*

#### Medication effectiveness

The last major subtheme was “Medication effectiveness” (Fig. 1). Pharmacists initiated a topic within this subtheme more often than patients and inquired whether patients experienced a beneficial effect of the medication and provided background information on specific medication, e.g. whether or not patients could experience an effect at all.

*Pharm19: “You also have to take tamsulosin, do you experience an effect? Pat54: “I don’t know really, I have to take a lot of different drugs, so I can’t tell if it’s beneficial.”*

Patients initiated topics within this subtheme to share their experiences with using medication and whether or not they see a positive effect from it in treating their health condition.

*Pat49: “Like I told before, I can sense it coming. So, that provides me with some time to get my inhaler. And it helps a lot.” Pharm18: “Yeah?” Pat49: “Yes, it helps me getting through it, especially on the warmer days. I really need my inhaler in the summer.”*

#### Less-discussed subthemes

The less discussed subthemes were “Hospital admission”, “Medication adherence” and “Beliefs about medication” (Fig. 1). Pharmacists dominated the initiation of the subtheme “Hospital admission” in which all topics concerning patients’ recent admission were discussed, such as the reason for admission and length of stay. Pharmacists used this question mostly as the opening question for the home visit.

*Pharm17: “Tell me, what was the matter? You were admitted to the hospital and what happened? Why were you admitted?” Pat47: “Well, I’ve been told that my symptoms suggested a hernia.”*

Furthermore, pharmacists asked patients which medication they were using besides the prescribed medication.

*Pharm14: “Do you use any over*-*the*-*counter drugs, ones purchased at the chemist maybe?” Pat35: No, I would never do that.” Pharm14: “No supplements either?” Pat35: “No, all those extra pills, I am not up for that. I think it is unnecessary.”*

The subtheme “Medication adherence” was initiated more often by pharmacists than by patients and involved pharmacists asking whether patients experienced adherence problems, for instance due to forgetfulness.

*Pharm16: “Do you forget to take your medication sometimes, a single tablet maybe?” Pat43: “No, never.” Pharm16: “So you are familiar with your daily regimen?” Pat43: “Yes, I prepare them all in advance.”*

The subtheme patients’ “Beliefs about medication” was initiated as often by pharmacists, e.g. to identify patients’ needs or expectations of their medication, as by patients who shared their general attitude towards medication. Furthermore, patients expressed specific concerns about using their medication.

*Pharm20: “Let’s see, what do you think about your medication?” Pat55: “Yes, I do experience the benefits, I mean, I have been taking them for a long time already and I’m still here!”*

*Pat12: “Well I’ve had a small hip fracture for which I took these pills. However, I try to minimize my intake because I worry that with prolonged use my body gets immune for it. And it’s the only painkiller I’m allowed to take!”*

#### Other themes

“Other” themes (Fig. 1) consisted mainly of patients’ dissatisfaction with the community pharmacy (e.g. pharmacy services or pharmacy stock), the health insurers (e.g. reimbursement issues), the hospital (e.g. transfer of information or waiting times) or the general practitioner (e.g. unwanted referral to hospital).

*Pat13: “You’ve always had a pharmacy delivery service, but nowadays you’re giving me a hard time.”*

*Pat6: “And then there is the health insurer who mess things up by deciding which medication I receive. Only the cheapest!”*

Furthermore, patients shared personal information, for instance about their grandchildren or the weather or asked study-related questions.

## Discussion

In this study we showed that administration and use of medication, e.g. regimen and actual drug-taking issues, knowledge gaps regarding medication and patients’ health were discussed most frequently, followed by medication logistics and medication effectiveness. Patients’ beliefs about medication and adherence were less frequently discussed. The pharmacist initiated the majority of these topics. Additional non-protocolled topics were scarce and consisted mainly of patient-initiated dissatisfaction regarding the community pharmacy or health insurers.

The most-discussed topics during the home visit consultation are in line with major challenges identified in previous studies and therefore crucial to address, e.g. patients’ lack of knowledge regarding their medication and medication regimen [3, 16]. The myriad of medication and clinical topics discussed during the home visits illustrate the rigor of the HomeCoMe protocol in identifying post-discharge drug-related problems (DRPs). The semi-structured protocol resulted in community pharmacists initiating the majority of topics. Pharmacists alternated between open-ended questions to increase patient engagement and more structured directive questions to gain information needed to identify possible DRPs. An active patient role is important as it results in greater satisfaction with the care they receive, a higher commitment to their treatment plans and a better understanding of their treatment, for instance [17, 18]. However, less-educated patients may find it difficult to ask the most relevant questions concerning their medication [19]. Furthermore, patients might not clearly express their information needs because they either assume that the pharmacist has told them everything or because they do not want to appear ignorant. Therefore, pharmacists need to empower patients in fulfilling that active role as it has been identified as a key factor to improve health outcomes [20]. On the other hand, pharmacists themselves embraced their counselling role by reinforcing hospital discharge information and elucidating possible existing or unresolved drug-related problems. Furthermore, as pharmacists were in the lead it should enable them to monitor the time spent on the home visit. The lack of dedicated time for pharmaceutical care was raised as a potential barrier for implementation in everyday community pharmacy practice, therefore monitoring time could possibly lower that barrier [21]. Other potential barriers for further implementation were the lack of a reimbursement fee, the inability of adopting the home visit into the current daily routine of the community pharmacist and inadequate skills in communication and pharmacotherapy of the community pharmacist [21].

Good communication skills are essential when providing patient-centred care to ensure patients’ understanding of their drug therapy and encourage adherence to their medication [6, 22]. Pharmacists need to be trained in applying general affective communicative strategies, listening and reflecting, and responding to uttered cues [23]. Combined with non-specific verbal behaviour techniques, such as social talk, these techniques are especially important in addressing patient concerns. They not only create a safe and inviting atmosphere between the pharmacist and patient but also encourage patients to disclose their emotions and concerns [23–25]. Furthermore, changing the consultation dynamic may also help; from a professional “coolness” approach at the beginning of the consultation to becoming warmer and avoiding non-verbal cut-offs at the end [24]. Incorporating more open-ended questions and follow-up questions throughout the home visit could increase the flexibility of the protocol and might invite patients to express their concerns [26].

It is important to discuss patient experiences, beliefs and adherence issues pro-actively, since not all patients might express these issues themselves. In this study, patients responded mainly with their dissatisfaction regarding health care professionals to these questions. Identifying and addressing these complaints is relevant, as it might facilitate patient participation and acceptance of pharmacists’ advices [27]. Performing the home visits in the privacy of patients’ own homes presents a unique opportunity to focus on these topics, in contrast to the turbulent and less private environment of the community pharmacy [22]. Therefore, to maximize the benefit of the pharmacist home visits, pharmacists should be provided with a more extensive training program focused on how they can explore these topics and which communication techniques they can use.

An important strength of this study was its large sample size, most likely ensuring data saturation. As this is the first study that qualitatively describes the topics during a post-discharge community pharmacist home visit, the results illustrate the post-discharge consequences for patients at readmission to primary care. Another strength is the substantial number of different pharmacists that conducted the home visits. Although they had the same training in advance, they differed in work experience thus minimizing possible biases such as when only a specific research pharmacist population was included. This increases the internal validity of this study. A limitation of this study is the use of a semi-structured protocol that resulted in pharmacists having less communicative freedom during the home visit. Therefore, mapping of the patient-pharmacist communication is possibly hampered as it expected to be substantially defined by the protocol. Furthermore, it resulted in pharmacists dominating the conversation. However, pharmacists provided room for patients to initiate those topics relevant to them in the introduction and concluding parts of the home visit. As patients mainly responded with dissatisfaction towards their health care providers, it is important to incorporate these topics within the protocol. Another possible limitation of this study was the use of audio recordings. This might have caused a behavioural change (Hawthorne effect) as the pharmacist and patient were aware that they were being recorded [28].

## Conclusion

Community pharmacists most frequently initiated practical issues regarding the administration and use of medication, followed by knowledge gaps regarding medication and patients’ health. Although included as a separate part of the protocol, pharmacists less frequently discussed patients’ medication and health beliefs. Additionally, patients initiated topics related to dissatisfaction with received care, which is important to address as it might facilitate patient participation and acceptance of pharmacists’ advices. Providing training programs for pharmacists to improve pharmacists’ communication skills in adopting general affective communicative strategies and non-specific verbal behaviour techniques during the consultations might improve pharmacist-patient interaction. These follow-up home visits provide an opportunity for community pharmacists to collaborate with patients to reinforce hospital discharge information in a safe environment for patients.
